# Optimal information encoding for multiple, simultaneously presented stimuli

**DOI:** 10.1186/1471-2202-13-S1-P17

**Published:** 2012-07-16

**Authors:** Jan Pieczkowski, Lawrence York, Jeanette Hellgren Kotaleski, Mark van Rossum

**Affiliations:** 1 Department of Computational Biology, CSC, Royal Institute of Technology, Stockholm, Sweden; 2 Department of Informatics, Edinburgh University, Edinburgh, EH8 9AB, UK; 3 Department of Neuroscience, Karolinska Institutet, Stockholm, Sweden

## 

Information in the brain is usually encoded in a way that distributes the activity over a population of neurons, referred to as *population coding*[1]. Population coding has been observed in almost all brain systems and renders the neural code robust, accurate, and failure resistant.

The coding of single stimuli in population codes is relatively well understood [2], and in particular the noise models, correlations, neural heterogeneity and links to psychophysics have been studied. However, the situation is much less clear when multiple stimuli are simultaneously encoded [3].

Theoretical studies (e.g., [4]) have thus far only examined linear supposition schemes that encode a probabilistic stimulus ensemble. However, experimental studies (c.f. [5], [6], [7]) suggest a non-linear encoding scheme using a maximum rule, where the response of a single neuron to a pair of stimuli equals the response to the constituent that on its own produces the maximum response, i.e.

We investigate the theoretical implications of these findings by comparing different encoding strategies and examine the decoding accuracy. The goal is to find the optimal encoding scheme for multiple stimuli.

We investigate the theoretical implications of these findings by comparing different encoding strategies and examine the decoding accuracy. The goal is to find the optimal encoding scheme for multiple stimuli.

In our current study, we focus on the simultaneous coding of visual stimuli representing overlapping movements of two groups of points in different directions. We investigate different ways of decoding these, among them a Maximum Likelihood decoder and estimate error rates made by these predictors, comparing to maximum rule to a linear rule.
